# Development a nomogram prognostic model for survival in heart failure patients based on the HF-ACTION data

**DOI:** 10.1186/s12911-024-02593-1

**Published:** 2024-07-19

**Authors:** Ting Cheng, Dongdong Yu, Jun Tan, Shaojun Liao, Li Zhou, Wenwei OuYang, Zehuai Wen

**Affiliations:** 1 Second Clinical College of Guangzhou University of Chinese Medicine, Guangzhou, China; 2 First Affiliated Hospital of Anhui University of Chinese Medicine, Hefei, China; 3 Guangdong Provincial Hospital of Chinese Medicine (Second Affiliated Hospital of Guangzhou University of Chinese Medicine), Guangdong Provincial Academy of Chinese Medical Sciences, Guangzhou, China; 4 Science and Technology Innovation Center of Guangzhou University of Chinese Medicine, Guangzhou, China

**Keywords:** Heart failure, HF-ACTION, Peak VO_2_, Survival, Prediction model

## Abstract

**Background:**

The risk assessment for survival in heart failure (HF) remains one of the key focuses of research. This study aims to develop a simple and feasible nomogram model for survival in HF based on the Heart Failure-A Controlled Trial Investigating Outcomes of Exercise TraiNing (HF-ACTION) to support clinical decision-making.

**Methods:**

The HF patients were extracted from the HF-ACTION database and randomly divided into a training cohort and a validation cohort at a ratio of 7:3. Multivariate Cox regression was used to identify and integrate significant prognostic factors to form a nomogram, which was displayed in the form of a static nomogram. Bootstrap resampling (resampling = 1000) and cross-validation was used to internally validate the model. The prognostic performance of the model was measured by the concordance index (C-index), calibration curve, and the decision curve analysis.

**Results:**

There were 1394 patients with HF in the overall analysis. Seven prognostic factors, which included age, body mass index (BMI), sex, diastolic blood pressure (DBP), exercise duration, peak exercise oxygen consumption (peak VO_2_), and loop diuretic, were identified and applied to the nomogram construction based on the training cohort. The C-index of this model in the training cohort was 0.715 (95% confidence interval (CI): 0.700, 0.766) and 0.662 (95% CI: 0.646, 0.752) in the validation cohort. The area under the ROC curve (AUC) value of 365- and 730-day survival is (0.731, 0.734) and (0.640, 0.693) respectively in the training cohort and validation cohort. The calibration curve showed good consistency between nomogram-predicted survival and actual observed survival. The decision curve analysis (DCA) revealed net benefit is higher than the reference line in a narrow range of cutoff probabilities and the result of cross-validation indicates that the model performance is relatively robust.

**Conclusions:**

This study created a nomogram prognostic model for survival in HF based on a large American population, which can provide additional decision information for the risk prediction of HF.

**Supplementary Information:**

The online version contains supplementary material available at 10.1186/s12911-024-02593-1.

## Introduction

Heart failure (HF) is a late stage of various heart diseases and has long been one of the diseases with high morbidity, high hospitalization rate, and high mortality rate [1]. There is an increasing burden of health and economic problems associated with HF in the world [2]. Due to the suddenness, variability, and complexity of the clinical course of HF, malignant events often occur. As a result, an increasing number of studies focused on risk prediction for the prevalence and mortality rate of HF and have created scoring systems or tools that include demographic factors, clinical factors, laboratory indicators, and biomarkers. Examples include the Barcelona bio-heart failure risk calculator (BCN bio-HF calculator) [3], the Heart Failure Scoring System (HFSS) [4], the risk calculator of Meta-Analysis Global Group in Chronic (MAGGIC) [5], and the risk model of the Seattle Heart Failure Model (SHFM) [6]. Currently, the indicators used in various HF prediction models are lacking a standardized predictor system. Despite varying degrees of validation, these risk prediction tools have not been widely applied in clinical practice. Due to the inconsistency of the study population, the differences in research methods, inherent variability in HF patients, and the complexity of the condition, the risk assessment of HF has always been a controversial result. So, the risk assessment regarding HF endpoint remains one of the key focuses of research.

Cardiopulmonary exercise testing (CPX) is recognized as one of the techniques used to assess the predictive ability of survival in HF patients. Some studies have confirmed the predictive value of CPX parameters such as peak oxygen uptake (VO_2_) and the regression slope relating minute ventilation to carbon dioxide output (VE/VCO_2_ slope) for HF patients [7, 8], however, its impact on HF is inconsistent and requires further substantial evidence. In recent years, the use of nomograms has become a new approach to predicting the risk of diseases [9], but there are few nomograms related to survival in chronic heart failure (CHF). Heart Failure-A Controlled Trial Investigating Outcomes of Exercise Training (HF-ACTION) study is a large-scale, multicenter, randomized controlled trial (RCT) funded by the National Institutes of Health (NIH). It is the largest study to date that includes CPX testing in patients with CHF [10]. The preliminary results of the HF-ACTION study indicated that peak VO_2_ is a significant predictor for the primary endpoint and all-cause mortality rate in patients with HF [11–14]. Therefore, we developed and validated a nomogram for predicting the performance of CPX parameters combined with others in estimating the risk of HF through retrospective analysis of the HF-ACTION database. This study aims to provide a simple HF risk assessment tool that is developed based on a large-scale American population.

## Patients and methods

### Study population

The HF-ACTION study was a multicenter RCT that enrolled 2331 CHF patients in the New York Heart Association (NYHA) classes II to IV and followed them for up to 4 years [15]. All of our data was extracted from the HF-ACTION database, which we obtained authorization to access after signing the National Heart, Lung, and Blood Institute (NHLBI) research materials distribution agreement. Individuals with missing values in independent variables and an unclear primary outcome will not be included in the analysis. A total of 1398 cases of HF met our inclusion criteria, and all eligible patients were randomly divided into a training cohort and a validation cohort at a ratio of 7:3. This study follows the Helsinki Declaration, and the decision to exempt it from review was given by the research ethics committee of Guangdong Provincial Hospital of Chinese Medicine (NO.YM-2021-066). Informed consent was waived by the same committee due to the study’s retrospective nature.

### Independent variables and primary outcome

The same variables were extracted from the HF-ACTION database for the training and validation cohorts. These variables include age, body mass index (BMI), race, sex, education, systolic blood pressure (SBP), diastolic blood pressure (DBP), resting heart rate, the type of etiology, best available baseline left ventricular ejection fraction (LVEF), baseline New York Heart Association (NYHA) class, Canadian Cardiovascular Society (CCS) angina class, medical history, exercise duration (CPX test), six-minute walk distance (SMWD), peak VO_2_, chronic medications, survival days, and vital status. The primary outcome is survival days in HF patients. Patients who were no-event at the end of the follow-up period or lost to follow-up were considered censored data and imputed using the last observation carried forward method.

### Nomogram construction

A training cohort was used to develop the nomogram. All extracted variables were entered into the univariate analysis. Univariate Cox regression model and Wald test were used to analyze significant prognostic-related variables with a *P* value < 0.1. Multivariate Cox regression analysis was performed using four methods: enter, forward, backward, and stepwise process. Redundant variables were eliminated through the backward stepwise process based on the minimum Akaike information criterion (AIC). A prognostic model was constructed based on the risk score calculated by the final Cox regression, and the results were visually displayed.

### Model performance and validation

In order to validate the performance of this prediction model, the C-index and the calibration curve were used. In general, the model performs better when the C-index is greater than 0.7 (ranging from 0 to 1.0) [16]. The area under the receiver operating characteristic (ROC) curve for 365- and 730-day survival is represented in graphical plots. Similarly, the closer the value of the area under the curve (AUC) is to 1, the higher the discrimination ability [17]. Calibration curves were plotted between the predicted survival probability and the actual survival proportion for 365- and 730-day survival. This nomogram was most accurate when calibration curves were closer to the reference line. Additionally, a decision curve analysis (DCA) was conducted to assess the net benefits of clinical decisions.

### Statistical analysis

SPSS 18.0 (IBM Corporation, Armonk, NY, USA) and R version 4.2.2 (The R Foundation for Statistical Computing, Vienna, Austria) were used for statistical analysis. Continuous variables were analyzed by mean and standard deviation (SD), while categorical variables were described by frequency and percentage. Univariable and multivariate Cox regression analysis was used to evaluate the independent prognostic factors among training cohort variables and analyze their impact on overall survival. The R packages ‘survival’ (version 3.4-0), ‘foreign’ (version 0.8–83), ‘rms’ (version 6.3-0), ‘survminer’ (version 0.4.9), and ‘survival ROC’ (version 1.0.3.1) were used for nomogram construction and evaluation [18]. Kaplan-Meier survival analysis and life table analysis were used to provide more information about the survival in HF. And evaluate the performance of the model using cross-validation with the ‘CoxBoost’ package. A two-tailed *P*-value of less than 0.05 was considered to be statistically significant.

## Results

### Characteristics of the demographic and clinical

**Fig. 1:**
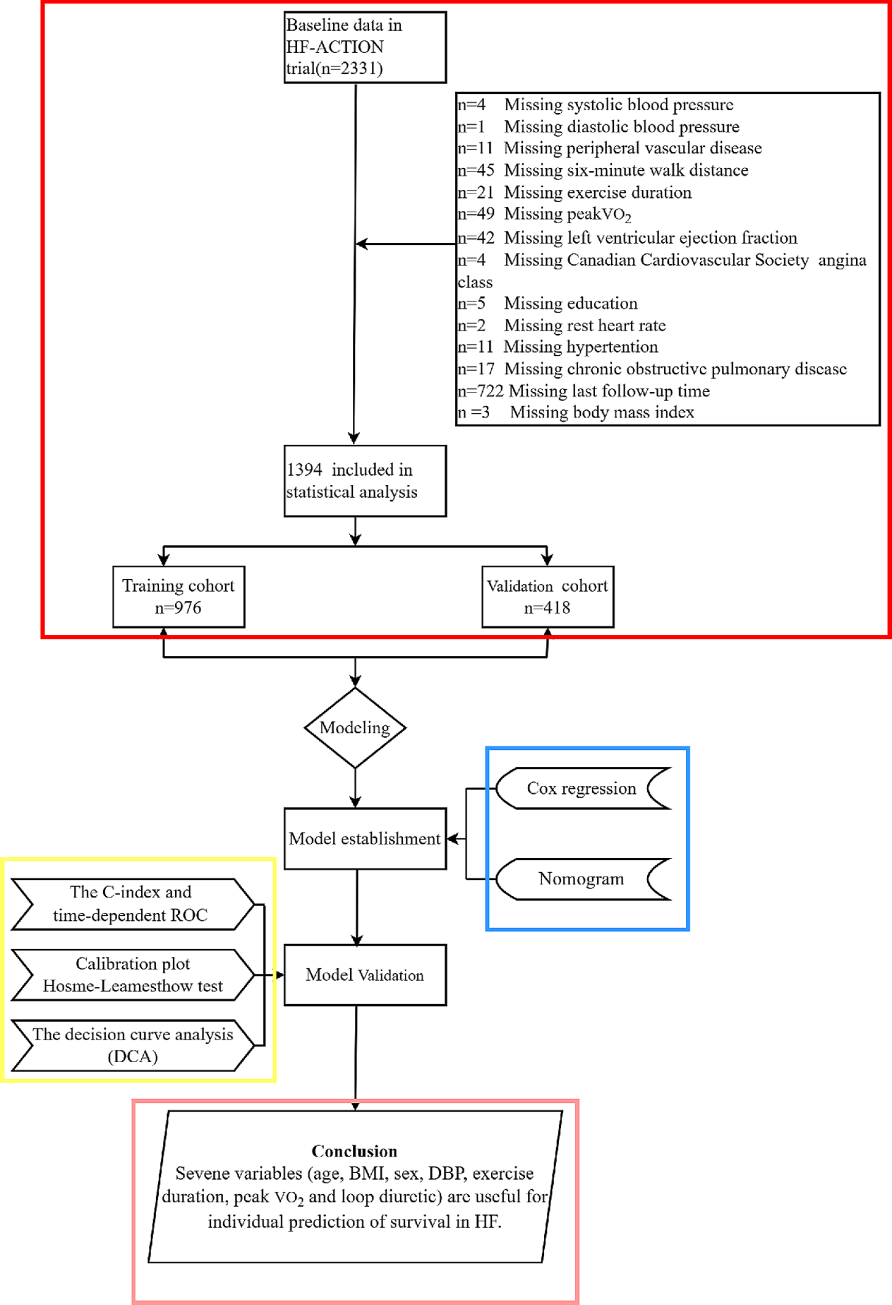
The flow diagram

The study flow chart is shown in Fig. 1 based on the HF-ACTION database. There were 976 patients with HF, which resulted in 223 deaths over a median follow-up duration of 806 days in the training cohort. A total of 418 cases of HF were included in the validation cohort, and 98 deaths occurred over a median follow-up of 853 days. The demographic characteristics of the training and validation cohorts are shown in Table 1.

**Table 1:** Demographic characteristics of the training and validation cohorts

Variables	Overall	Training cohort	Validation cohort
No. of patients	1394	976	418
Age (year)	58.90 ± 12.92	58.61 ± 13.40	59.01 ± 12.87
BMI (kg/m^2^)	30.98 ± 7.06	30.98 ± 6.95	30.99 ± 7.31
Sex			
Male	994(71.31%)	693(71.00%)	301(72.01%)
Female	400(28.69%)	283(29.00%)	117(27.99%)
Race			
Black	481(34.51%)	328(33.61%)	153(36.60%)
White	820(58.82%)	585(59.94%)	235(56.22%)
African American	70(5.02%)	47(4.82%)	23(5.50%)
Others	23(1.65%)	16(1.64%)	7(1.67%)
Education			
< High school	159(11.41%)	115(11.78%)	44(10.53%)
> = High school	1235(88.59%)	861(88.22%)	374(89.47%)
SBP(mmHg)	113.66 ± 18.19	113.94 ± 18.47	113.00 ± 17.50
DBP(mmHg)	70.07 ± 11.35	70.05 ± 11.42	70.13 ± 11.19
Rest heart rate(bpm)	70.94 ± 11.31	70.69 ± 11.05	71.53 ± 11.88
Etiology			
Ischemic	734(52.65%)	511(52.36%)	223(53.35%)
Non-ischemic	660(47.35%)	465(47.64%)	195(46.65%)
LVEF	24.90 ± 7.51	25.28 ± 7.62	24.02 ± 7.20
SMWD (meters)	359.70 ± 102.73	359.15 ± 103.99	360.96 ± 99.83
Peak VO_2_(mL/kg/min)	14.44 ± 4.52	14.37 ± 4.50	14.59 ± 4.56
Exercise duration (minutes)	9.47 ± 3.84	9.38 ± 3.79	9.70 ± 3.95
NYHA class			
II	852(61.12%)	597(61.17%)	255(61.00%)
III	525(37.66%)	367(37.60%)	158(37.80%)
IV	17(1.22%)	12(1.23%)	5(1.20%)
CCS angina class			
0	1150(82.50%)	800(81.97%)	350(83.73%)
1	130(9.33%)	89(9.12%)	41(9.81%)
2	114(8.18%)	87(8.91%)	27(6.46%)
Medical History			
Angina			
no	1016(72.88%)	715(73.26%)	301(72.01%)
yes	378(27.12%)	261(26.74%)	117(27.99%)
Myocardial Infarction			
no	787(56.46%)	560(57.38%)	227(54.31%)
yes	607(43.54%)	416(42.62%)	191(45.69%)
Hypertention			
no	522(37.45%)	381(39.04%)	141(33.73%)
yes	872(62.55%)	595(60.96%)	277(66.27%)
COPD			
no	1228(88.09%)	849(86.99%)	379(90.67%)
yes	166(11.91%)	127(13.01%)	39(9.33%)
Diabetes			
no	928(66.57%)	642(65.78%)	286(68.42%)
yes	466(33.43%)	334(34.22%)	132(31.58%)
Depression			
no	1105(79.27%)	775(79.41%)	330(78.95%)
yes	289(20.73%)	201(20.59%)	88(21.05%)
PVD			
no	1302(93.40%)	910(93.24%)	392(93.78%)
yes	92(6.60%)	66(6.76%)	26(6.22%)
Stoke			
no	1245(89.31%)	879(90.06%)	366(87.56%)
yes	149(10.69%)	97(9.94%)	52(12.44%)
Chronic Medications			
ACEI			
no	366(26.26%)	251(25.72%)	115(27.51%)
yes	1028(73.74%)	725(74.28%)	303(72.49%)
Beta blocker			
no	83(5.95%)	56(5.74%)	27(6.46%)
yes	1311(94.05%)	920(94.26%)	391(93.54%)
Loop diuretic			
no	289(20.66%)	203(20.80%)	85(20.33%)
yes	1106(79.34%)	773(79.20%)	333(79.67%)
Digoxin			
no	747(53.59%)	530(54.30%)	217(51.91%)
yes	647(46.41%)	446(45.70%)	201(48.09%)

BMI: body mass index; SBP: Systolic blood pressure; DBP: Diastolic blood pressure; peak VO_2_: peak oxygen uptake; LVEF: left ventricular ejection fraction; SMWD: Six-minute walk distance; COPD: chronic obstructive pulmonary disease; PVD: peripheral vascular disease; ACEI: angiotensin-converting enzyme inhibitor

### Training cohort independent prognostic factors

A Cox proportional hazards model was used to assess the independent prognostic factors among training cohort variables, and the results were shown in Table 2. In the univariate analysis, age, BMI, sex, DBP, baseline NYHA class, SMWD, exercise duration, peak VO_2_, best available baseline LVEF, types of etiology, history of myocardial infarction, peripheral vascular disease (PVD), chronic obstructive pulmonary disease (COPD), diabetes and loop diuretics were revealed to be significant correlating variables for survival in HF (*P* < 0.05). The final model after multivariate analysis comprises exercise duration, peak VO_2_, age, BMI, gender, DBP, and loop diuretics, which were selected from 15 meaningful variables in the univariate analysis using the method backward.

**Table 2:** Results of univariable and multivariate Cox proportional hazards regression analysis for overall survival

Variables	Univariable analysis			Multivariate analysis		
	HR	95% CI	*P*	HR	95% CI	*P*
Age (year)	0.96	1.03–1.05	< 0.001	1.01	1.00-1.03	0.041
BMI (kg/m^2^)	1.03	0.96-1.00	0.015	0.98	0.96–1.01	0.153
Sex	1.73	0.42–0.80	0.001	0.47	0.34–0.66	< 0.001
Race	0.96	0.84–1.30	0.679			
Education	1.08	0.85–1.02	0.111			
SBP	1.01	0.99–1.00	0.126			
DBP	1.02	0.97–1.00	0.006	0.99	0.98–1.00	0.082
Rest heart rate (bpm)	1.00	0.99–1.01	0.922			
Etiology	1.45	0.53–0.90	0.006			
LVEF	1.02	0.96–1.00	0.022			
SMWD (meters)	1.00	0.99–1.00	< 0.001			
Peak VO_2_(mL/kg/min)	1.16	0.83–0.89	< 0.001	0.91	0.87–0.97	0.001
Exercise duration (minutes)	1.18	0.81–0.88	< 0.001	0.91	0.85–0.97	0.003
NYHA class	0.59	1.33–2.15	< 0.001			
CCS angina class	1.18	0.67–1.07	0.173			
Angina	1.10	0.68–1.23	0.538			
Myocardial Infarction	0.71	1.08–1.83	0.011			
Hypertension	1.07	0.72–1.23	0.637			
COPD	0.57	1.25–2.45	0.001			
Diabetes	0.66	1.16–1.98	0.002			
Depression	1.21	0.59–1.16	0.268			
PVD	0.39	1.75–3.78	< 0.001			
Stroke	0.73	0.92–2.05	0.124			
ACEI	1.06	0.70–1.27	0.691			
Beta blocker	1.15	0.51–1.47	0.595			
Loop diuretic	0.56	1.23–2.63	0.002	1.43	0.98–2.10	0.066
Digoxin	0.86	0.89–1.51	0.271			

HR: Hazard ratio; CI: Confidence Intervals; BMI: body mass index; SBP: Systolic blood pressure; DBP: Diastolic blood pressure; peak VO_2_: peak oxygen uptake; LVEF: left ventricular ejection fraction; SMWD: Six-minute walk distance; COPD: chronic obstructive pulmonary disease; PVD: peripheral vascular disease; ACEI: angiotensin-converting enzyme inhibitor

### Establish the prognostic nomogram model

**Fig. 2:**
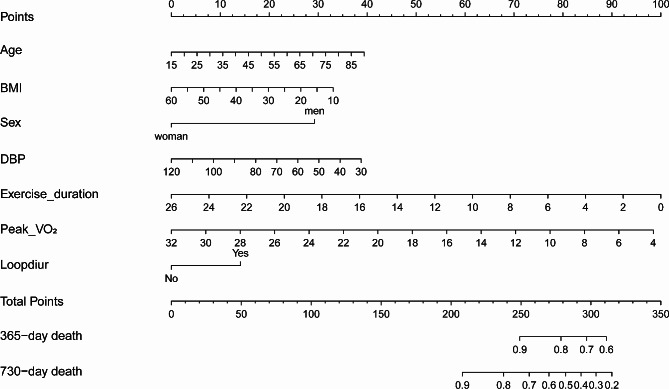
Nomogram for predicting overall survival in patients

Finally, seven variables (age, BMI, sex, DBP, exercise duration, peak VO_2_, and loop diuretic) were selected to develop the nomogram model. The final model’s AIC is 2724.894, which is the smallest among the four methods of enter, forward, backward, and stepwise process. Each variable was assigned a point score from 0 to 100 in the nomogram for all-cause mortality (Fig. 2). Peak VO_2_ and exercise duration showed the greatest influence on prognosis, with the highest score can reach 100. It is noteworthy that age, BMI, and DBP contributed approximately equally to survival predictions. By summarizing each variable’s score for a patient on the uppermost rule, we calculated the total number of points. The corresponding predicted probability of mortality could be found on the lowest rule. The survival individual risk scores at 365- and 730-days could be determined. The Kaplan-Meier (K-M) curves and life table analysis can provide more information about the nomogram (Fig. 1 and Table l, 2 in supplementary materials).

### Model performance and validation of the nomogram

**Fig. 3:**
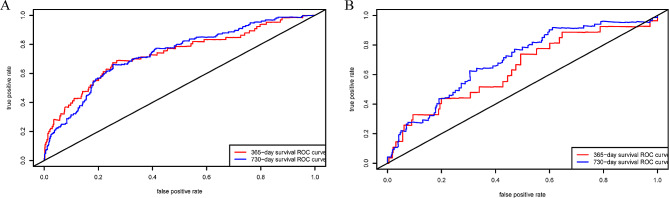
AUC represents the model’s discriminatory ability. (**A**) shows the AUC of the training cohort, and (**B**) shows the AUC of the validation cohort

**Fig. 4:**
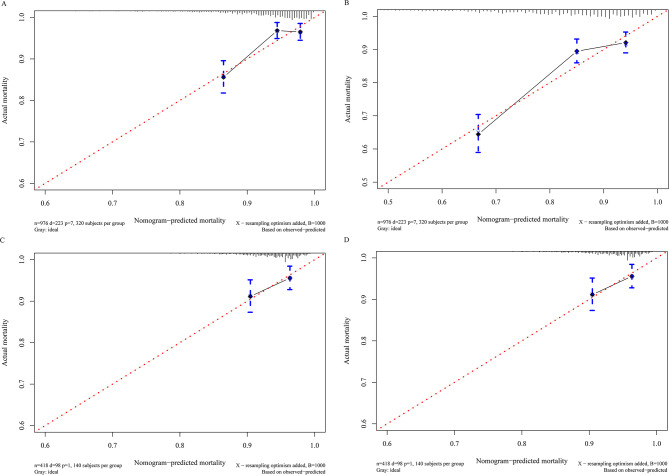
Calibration curve shows the degree of consistency between the predicted probability and observed probability (Hosmer-Lemeshow test indicates goodness-of-fit, *P* > 0.05). **A** and **B** shows the 365-and 730-day calibration curve of the training cohort, and C and D shows the 365- and 730-day calibration curve of the validation

**Fig. 5:**
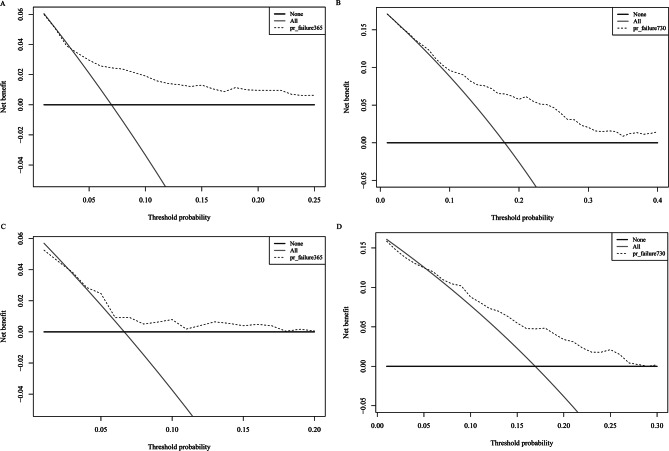
DCA represents the clinical net benefit of the nomogram (nomogram is represented by the dotted line). **A** and **B** shows the 365-and 730-day DCA of the training cohort, and **C** and **D** shows the 365- and 730-day DCA of the validation

The C-index for the established model was 0.715 (95% confidence interval [CI]: 0.700, 0.766) in the training cohort, and the C-index was 0.662 (95% CI: 0.646, 0.752) in the validation cohort. The 365- and 730-day time-dependent ROC curves of the models were shown in Fig. 3. The areas under the ROC curve (AUC) of the training group and the validation group were (0.731, 0.734) and (0.640, 0.693), respectively, for 365- and 730-day survival. Calibration plots at 365- and 730-day survival showed a good correlation between predicted survival probability and actual observation in both cohorts (Fig. 4). The decision curve analysis (DCA) showed that our nomogram model provided a net benefit in a narrow threshold probability when compared with either treating all or treating none (Fig. 5). The result of cross-validation indicates that the model performance is relatively robust (Table 1 in supplementary materials).

## Discussion

The study retrospectively analyzed the HF-ACTION dataset of a large population in the United States. A nomogram model containing seven parameters (age, BMI, sex, DBP, exercise duration, peak VO_2_, and loop diuretic) was developed and internally validated. Although the model only showed net benefits within a critical probability range, it demonstrated good predictive ability consistency and calibration in both the training and validation sets. This will provide additional decision information for the risk prediction of HF.

The prognosis of HF has consistently been a central focus and challenge in research, emphasizing the critical importance of early screening and intervention. Factors such as age, gender, BMI, and DBP are acknowledged as independent variables influencing the survival of CHF patients in our study, aligning with findings from prior studies. A study confirmed that the primary factors (age, SBP, potassium, sex, and acute myocardial infarction) can be utilized to predict the survival of individuals with acute heart failure (AHF) [19]. The Acute Study of Nesiritide in Decompensated Heart Failure (ASCEND-HF) has released a simplified scoring system consisting of 5 variables (age, low SBP, low sodium, high BUN, dyspnoea at rest) for the purpose of categorizing early and long-term mortality in patients with decompensated HF [20]. The regression model of the Acute Decompensated Heart Failure National Registry (ADHERE) can utilize age, blood pressure, and biomarkers to forecast the likelihood of hospitalization mortality in patients with HF [21]. The mortality rate of HF patients in real medical settings is higher than in clinical trials, especially among the elderly [22]. As age increases, changes in the body, such as decreased myocardial elasticity, increased blood pressure, and vascular structural changes, can affect heart function, worsen, and impact survival in HF [23]. Additionally, elderly individuals may experience exacerbating factors such as malnutrition, weakness, decreased physical activity levels, poor medication adherence, lack of healthcare opportunities, cognitive impairments, diabetes, coronary artery disease, and hypertension, all of which can worsen and reduce survival rates in HF [24, 25]. Some studies suggested that male is independently associated with the risk of HF but not with combined endpoint and all-cause mortality rates. Although men are more likely to die from HF with preserved ejection fraction (HFpEF), women are more likely to die from HF with reduced ejection fraction (HfpEF), indicating that the mechanisms behind these associations remain unclear [26]. However, compared to men, women have a lower quality of life and more significant functional impairment in HF [27]. Our research findings similarly suggest that the overall prognosis after being diagnosed with HF may be better for women than for men, which could be related to the impact of pregnancy on the cardiovascular system [28]. It is also possible that differences between male and female patients with HF in terms of etiology, cardiac remodeling patterns, and other factors can affect their prognosis [29]. A fall in DBP has been shown to reduce coronary perfusion pressure, resulting in ischemia and myocardial damage [30, 31]. One study has found that too high or too low DBP is associated with an increased risk of mortality in HF [32]. Also, there was an increased risk of morbidity and mortality in patients at high cardiovascular risks with DBP < 70 mmHg [33]. Another study proposed that a DBP ≤ 60 mmHg is a risk factor for the mortality of HF, and patients with higher DBP at rest or exceeding specific DBP during exercise have better prognoses [34]. The above is consistent with the findings of this study. The 2020 International Society of Hypertension (ISH) guidelines recommended that the target DBP for patients with HF should be between 70 and 80 mmHg [33]. Therefore, proper blood pressure control is crucial for the survival of HF patients.

Peak VO_2_ is an index used to measure exercise capacity and has long been considered a powerful predictor of survival in HF patients, affecting survival assessment and decisions regarding cardiac transplantation [35–37]. Notably, peak VO_2_ may be influenced by non-cardiac factors such as age, gender, endurance training, anemia, muscle imbalance, and body composition, which may lead to misleading prognostic information [38]. The FIT-CPX Project observed survival differences in gender based on peak VO_2_ and ventilation to the volume of carbon dioxide produced (VE-VCO_2_) slope [39]. The longitudinal analysis of the early HF-ACTION study also confirmed that decreased exercise capacity and peak VO_2_ are independent predictors of clinical endpoints. Moderate exercise training is safe and effective for patients with HF. The early HF-ACTION trial also found that exercise training can somewhat reduce cardiovascular mortality and hospitalization rates [14]. The duration of exercise may affect the survival rate by enhancing myocardial contractility, improving myocardial metabolism, improving vascular function, regulating muscle sympathetic nerve activity, and promoting cardiac remodeling. However, the specific mechanism is unclear [40, 41]. Additionally, although there have been some studies evaluating the impact of cardiopulmonary parameters on the prognosis of HF patients, for example, the Henry Ford Hospital Cardiopulmonary Exercise Testing (FIT-CPX) Project [39], the PROBE study [37] and the HYPERHF study [42], however, it only calculates the discrimination of the overall sample results, without conducting calibration curve and DCA analysis, let alone verification. Therefore, our study focused on the impact of CPX parameters on the survival of HF patients through modeling methods, providing additional decision-making information for evaluating how to regulate cardiovascular function parameters through exercise training.

Loop diuretics are the cornerstone of the treatment of congestion in HF patients, and the effect of loop diuretics on long-term prognosis remains uncertain [43]. The use of loop diuretics in HF patients has been a topic of debate, with some studies suggesting a potential negative impact on survival. Eshaghian S et al. [44] found a dose-dependent association between loop diuretic use and impaired survival in patients with advanced HF. Similarly, Dini FL et al. [45] reported that increasing doses of furosemide, a commonly used loop diuretic, were associated with reduced survival in outpatients with CHF. However, Hasselblad V et al. [46] found that higher in-hospital diuretic doses were not associated with increased mortality in hospitalized HF patients. More recently, Faselis C et al. [47] reported that loop diuretic use was associated with better 30-day clinical outcomes in older patients with HF. These conflicting findings highlight the need for further research to clarify the impact of loop diuretics on survival in HF patients. Our findings indicate that loop diuretics contribute minimally to poor prognosis in heart failure within the model. Further research is needed to establish their long-term impact on heart failure prognosis.

The nomogram developed in this study offers several clinical advantages. First, the HF-ACTION database is a large multi-center RCT, which ensures a reliable data source of large samples. Second, The determined parameters can be easily measured and obtained through non-invasive examinations, which can be easily implemented in clinical practice. Additionally, the model integrates the predictive performance of cardiopulmonary parameters, providing additional evidence for the predictive ability of cardiopulmonary parameters in heart failure prognosis. Moreover, the good performance of the model, as evidenced by the C-index and AUC values, suggests that it can reliably predict survival in HF patients. This can facilitate early identification of high-risk patients and allow for timely interventions to improve clinical outcomes.

Despite the advantages above, this study still has limitations that are currently difficult to overcome. Firstly, due to the difficulty in obtaining similar data from other databases, the results have not been externally validated in different source databases, which may limit its generalizability. Similarly, the primary outcome is survival due to the limitation of research data to the HF-ACTION database. Still, the study does not delve into the quality of life, functional outcomes, or other outcomes (such as readmission rate) that are critical for HF patients. Thirdly, the study still needs to translated the nomogram into a practical program or app, posing specific challenges in its clinical workflow usability. Further research required to validate the model’s broad applicability and translate the research results into practical and high-performability decision tools. Furthermore, we need to delve into the potential interactions and synergistic effects among the seven prognostic factors, which will contribute to a more comprehensive understanding of the prognosis of HF. Finally, we must acknowledge that the DCA curve only shows net gain within a narrow range of critical probabilities, indicating the need for careful consideration in clinical application processes.

## Conclusions

This study created an internal validated nomogram prognostic model, which includes age, BMI, gender, DBP, exercise duration, peak VO_2_, and loop diuretic use, for survival in HF patients based on a large American population. This model may provide additional decision information for the risk prediction of HF.

### Electronic supplementary material

Below is the link to the electronic supplementary material.

Supplementary Material 1

## Data Availability

The datasets used and analyzed during the current study are available from the corresponding author upon reasonable request. Additionally, BioLINCC (https://biolincc.nhlbi.nih.gov/update/) can be used to apply for HF-ACTION data online once your research plan has been prepared and approved.

## Abbreviations

HF: heart failure
HF-ACTION: Heart Failure-A Controlled Trial Investigating Outcomes of Exercise TraiNing
BMI: body mass index
DBP: diastolic blood pressure
peak VO_2_: peak exercise oxygen consumption
BCN bio-HF calculator: Barcelona bio-heart failure risk calculator
HFSS: Heart Failure Scoring System
MAGGIC: Meta-Analysis Global Group in Chronic
SHFM: Seattle Heart Failure Model
CPX: cardiopulmonary exercise testing
CHF: chronic heart failure
NIH: National Institutes of Health
NYHA: New York Heart Association
NHLBI: National Heart, Lung, and Blood Institute
SBP: systolic blood pressure
LVEF: left ventricular ejection fraction
CCS: Canadian Cardiovascular Society
SMWD: six-minute walk distance
AIC: Akaike information criterion
SD: standard deviation
PVD: peripheral vascular disease
GWTG-HF: Get With The Guidelines-Heart Failure
ASCENDHF: Acute Study of Nesiritide in Decompensated Heart Failure
CI: confidence interval
AUC: area under the ROC curve
DCA: decision curve analysis
ADHERE: Acute Decompensated Heart Failure National Registry
FIT-CPX: The Henry Ford Hospital Cardiopulmonary Exercise Testing
PROBE: (P)e(R)i(O)dic (B)reathing during (E)xercise
HYPERHF: HYPertrophicExercise-derivedRiskHF
HFpEF: heart failure with preserved ejection fraction
HfpEF: heart failure with reduced ejection fraction
ISH: Inter­national Society Hypertension
