# Supplementary material for: Development a nomogram prognostic model for survival in heart failure patients based on the HF-ACTION data
Source: BMC Med Inform Decis Mak. 2024 Jul 19;24:197. doi: 10.1186/s12911-024-02593-1 (PMC11264587; doi:10.1186/s12911-024-02593-1)
Supplement: Supplementary file 1 — Supplementary Material 1 [file 12911_2024_2593_MOESM1_ESM.docx]

**Supplementary Table S1 Comparison of cross validation results and nomogram model results.**

| Variables | Multivariate analysis |  | Cross-validation |
| --- | --- | --- | --- |
|  | HR |  | HR |
| Age | 1.01 |  | 1.58 |
| BMI | 0.98 |  | 0.82 |
| Sex | 0.47 |  | 0.74 |
| DBP | 0.99 |  | 0.89 |
| Peak VO_2_ | 0.91 |  | 0.84 |
| Exercise duration | 0.91 |  | 0.60 |
| Loop diuretic | 1.43 |  | 1.11 |

HR: Hazard ratio; BMI: body mass index; DBP: Diastolic blood pressure; Peak VO_2_: peak oxygen uptake; the results with cross-validation using the CoxBoost package.


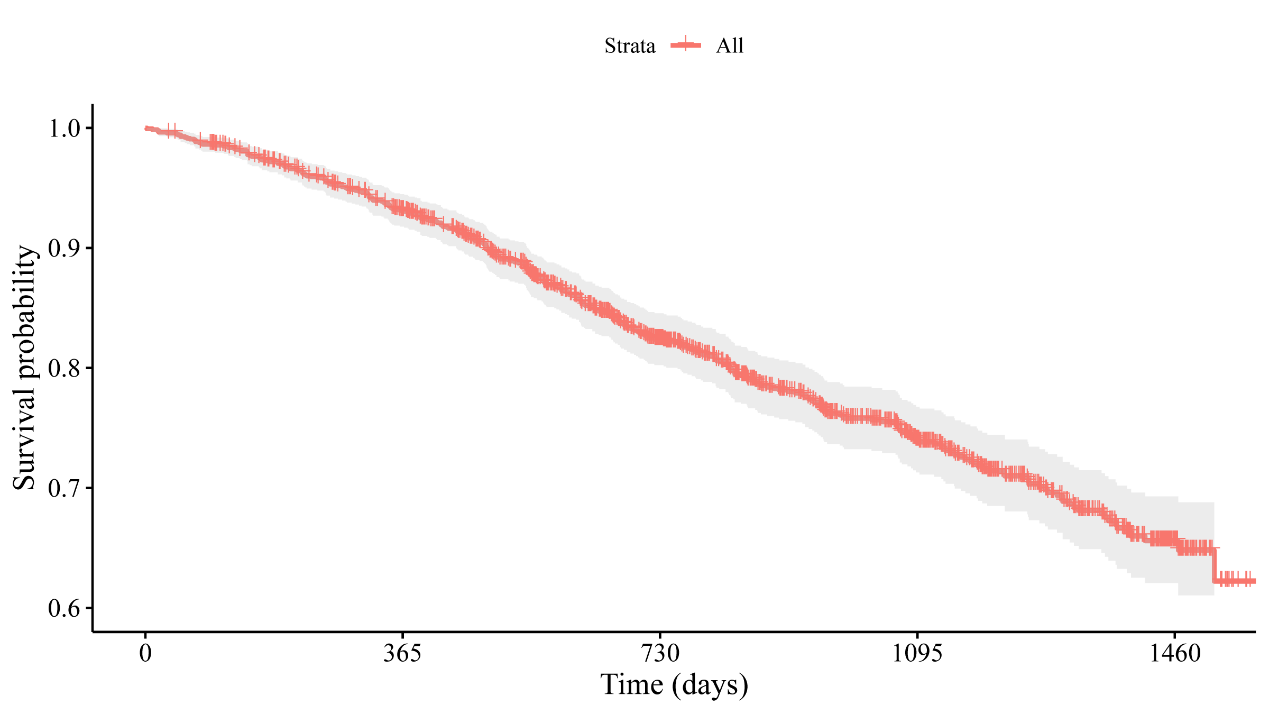


**Supplementary Fig S1 Cumulative incidence of death events.**

**Supplementary Table S2 The result of life table analysis.**

| Interval Start Time | Number Entering Interval | Number Withdrawing during Interval | | Number Exposed to Risk | Number of Terminal Events | Proportion Terminating | Proportion Surviving | Cumulative Proportion Surviving at End of Interval | Std. Error of Cumulative Proportion Surviving at End of Interval | Probability Density | Std. Error of Probability Density | Hazard Rate | Std. Error of Hazard Rate |
| --- | --- | --- | --- | --- | --- | --- | --- | --- | --- | --- | --- | --- | --- |
| 0 | 1394 | | 89 | 1349.500 | 93 | 0.07 | 0.93 | 0.93 | 0.01 | 0.000 | 0.000 | < 0.001 | 0.000 |
| 365 | 1212 | | 293 | 1065.500 | 123 | 0.12 | 0.88 | 0.82 | 0.01 | 0.000 | 0.000 | < 0.001 | 0.000 |
| 730 | 796 | | 268 | 662.000 | 67 | 0.10 | 0.90 | 0.74 | 0.01 | 0.000 | 0.000 | < 0.001 | 0.000 |
| 1095 | 461 | | 316 | 303.000 | 36 | 0.12 | 0.88 | 0.65 | 0.02 | 0.000 | 0.000 | < 0.001 | 0.000 |
| 1460 | 109 | | 106 | 56.000 | 2 | 0.04 | 0.96 | 0.63 | 0.02 | 0.000 | 0.000 | < 0.001 | 0.000 |
| 1825 | 1 | | 1 | 0.500 | 0 | 0.00 | 1.00 | 0.63 | 0.02 | 0.000 | 0.000 | < 0.001 | 0.000 |
